# Integration of Geriatric Psychiatry and Geriatrics: Enhancing Mental Health Assessment in the Elderly

**DOI:** 10.1192/j.eurpsy.2024.1313

**Published:** 2024-08-27

**Authors:** A. F. Borges, V. Domingues

**Affiliations:** Serviço de Psiquiatria e Saúde Mental, Centro Hospitalar de Leiria, Leiria, Portugal

## Abstract

**Introduction:**

The ageing population presents complex clinical challenges, particularly in the realm of mental health among the elderly. The intersection of geriatric psychiatry and geriatrics plays a critical role in providing a holistic and comprehensive approach to addressing psychiatric issues in older adults. This abstract explores the integration of these two disciplines and their significance in elderly mental health care.

**Objectives:**

This study aims to underscore the benefits of collaboration between geriatric psychiatry and geriatrics while highlighting areas of intersection. These areas include the assessment of medical and psychiatric comorbidities, the management of neuropsychiatric disorders, and the promotion of healthy ageing, both physically and mentally.

**Methods:**

We comprehensively reviewed the literature, encompassing research studies, case reports, clinical guidelines, and reports published in the last 10 years. The research was conducted on medical databases, including PubMed, Medline, and specialized sources in gerontology.

**Results:**

Effective integration between geriatric psychiatry and geriatrics provides a more comprehensive and patient-centred approach to addressing the mental health needs of the elderly. This includes enhanced assessment and treatment of a wide range of psychiatric conditions commonly found in older adults, such as cognitive disorders (including dementia and mild cognitive impairment), mood disorders (including depression and bipolar disorder), anxiety disorders, psychotic disorders, and substance use disorders. Additionally, the collaboration ensures a better understanding of the complex interplay between physical and mental health in the ageing population. The integration approach also encompasses the management of neuropsychiatric symptoms associated with various medical conditions common in older adults, such as delirium and behavioural disturbances in dementia. This coordinated care extends to the judicious use of psychotropic medications, considering the unique pharmacokinetics and pharmacodynamics in the elderly population, with a focus on minimizing adverse effects and drug-drug interactions. Furthermore, promoting emotional well-being and preventing mental illnesses emerge as critical areas of collaboration between these disciplines. Strategies for achieving this goal include psychoeducation, lifestyle interventions, and fostering a supportive environment for the elderly.

**Conclusions:**

In summary, the collaboration between geriatric psychiatry and geriatrics is crucial for addressing the complex mental health needs of the elderly, providing patient-centred care, and optimizing resources. This integrated approach is essential in ensuring the well-being of older adults, emphasizing a holistic, multidisciplinary approach to mental health issues in this population.

**Disclosure of Interest:**

None Declared

